# Can preoperative ureteral stents reduce the incidence of ureteral stricture after radiotherapy in patients with cervical cancer?

**DOI:** 10.1186/s12894-022-01029-0

**Published:** 2022-07-18

**Authors:** Liang Liu, Chunhong Yu, Fuzhen Sun, Tao Yang, Dong Wei, Gang Wang, Shoubin Li, Junjiang Liu

**Affiliations:** 1 Department of Urology, Hebei General Hospital, Shijiazhuang, China; 2 Department of Urology, Baoding NO.1 Central Hospital, Baoding, China; 3 Department of Medical Examination Center, Hebei General Hospital, Shijiazhuang, China

**Keywords:** Ureteral Stents, Cervical neoplasms, Radiotherapy, Ureteral stricture, Preoperative stent placement

## Abstract

**Objective:**

To determine the impact of preoperative stent placement on postradiotherapy stricture rate in patients with cervical cancer after radical resection.

**Methods:**

This study was a retrospective analysis of data collected from 55 cervical cancer patients treated with radiotherapy between June 2016 and June 2020. Patients were divided into the stent and control groups. After 3 months, the stricture rate and the complications related to stent placement between the two groups were compared.

**Results:**

There were 12 (46.2%) and 10 (34.5%) cases of ureteral stricture in the stent (n = 26) and control (n = 29) groups, respectively, three months after the end of radiotherapy. The incidence rates of ureter stricture in the two groups were not significantly different (*P* = 0.378). Moreover, there were 20 units (38.5%) and 15 units (25.9%) ureteral strictures in the stent and control groups, respectively. No significant difference in the incidence rates of ureteral strictures was found between the two groups (*P* = 0.157). There were 13 (50.0%) and 10 (34.5%) cases of ureteral stricture in the stent (n = 26) and control (n = 29) groups, respectively, six months after the end of the radiotherapy. The incidence rates of ureter stricture in the two groups were not significantly different (*P* = 0.244). Moreover, there were 21 units (40.4%) and 15 units (25.9%) ureteral strictures in the stent and control groups, respectively. No significant difference in the incidence rates of ureteral strictures was found between the two groups (*P* = 0.105). Complications related to stent placement such as urinary tract infections and bladder irritation were statistically significant (*P* = 0.006 and *P* = 0.036) between the two groups; while the other complications were not significantly different (*P* = 0.070, *P* = 0.092 and *P* = 0.586).

**Conclusions:**

Ureteral stents may not reduce the incidence of ureteral stricture after radiotherapy in patients with cervical cancer. The stent needs to be replaced regularly, and the complications related to stent placement may occur at any time. Thus, preoperative stent placement should be cautious for the clinical management of cervical cancer patients treated with postoperative radiotherapy.

## Introduction

The most common treatment for cervical cancer is surgery. Adjuvant radiotherapy may be given to patients if the risk factors affecting prognosis are found pathologically [1]. Radiotherapy has a definite effect on cervical cancer, but the incidence of ureteral strictures caused by this treatment is high (up to 16%) [2–4]. Ureteral stricture not only causes dilatation of the upper urinary tract and impairs renal function, but it significantly reduces the quality of life and affects the follow-up treatment of the primary disease.

It has been reported in the literature that the probability of ureteral injury to laparoscopic radical resection of cervical cancer is about 0.2–1.5% [5, 6]. Therefore, to avoid ureteral injury, some scholars choose to reserve ureteral stents during radical operation. This may reduce the incidence of ureteral stricture after radiotherapy. However, it remains inconclusive whether preoperative stent placement can reduce the occurrence of ureteral stricture.

In this study, we selected cervical cancer patients treated with adjuvant radiotherapy after laparoscopic radical resection as our research subjects. We aimed to explore whether preoperative stent placement can reduce the incidence of ureteral stricture in these patients.

## Materials and methods

### General information

A total of 55 patients with stage Federation of Gynecology and Obstetrics (FIGO) I–III who were treated with adjuvant radiotherapy after radical resection of cervical cancer between June 2016 to June 2020 were selected [7]. The patients were divided into two groups: (i) stent group (n = 26) and (ii) control group (n = 29). The stent group was stent placement before radical hysterectomy, and the stents were removed at 3 months after radiotherapy. The control group was no stent placement before radical hysterectomy.

The study protocol was approved by the ethics committee of the Hebei General Hospital, and was conducted in accordance with the principles of the Declaration of Helsinki and Good Clinical Practice Guidelines. Informed consent forms were signed by all the patients who accepted the above treatment.

### Inclusion and exclusion criteria

The inclusion criteria were as follows: (i) cervical cancer was diagnosed according to the FIGO pathological staging I–III; (ii) patients completed the full course of radiotherapy in Hebei General Hospital and reviewed regularly; (iii) the clinical data was complete, with no loss to follow-up; (iv) examined for ureteral stricture at preoperative and after radiation; and (v) the follow-up time was at least 6 months. Patients were excluded if they (i) did not receive full treatment; (ii) had distant metastasis or neurological complications; (iii) failed to cooperate; and (iv) had a previous history of ureteral stricture or related surgical procedures.

### Treatment

#### Placement of ureteral stents

Patients in the stent group were treated in the lithotomy position, and the cystoscope was placed under anesthesia, while those in the control group did not undergo stent placement.

#### Surgery

Patients in the stent group were treated in the lithotomy position, and the cystoscope was placed under anesthesia. The zebra guidewire was inserted into the renal pelvis through the ureteral orifice. Then, Contour™ ureteral stents (double-J, 6F, length = 24 cm, open-ended, model: M0061802220; Boston Scientific Corp., USA) were inserted retrogradely into the guidewire. A kidney, ureter, and bladder (KUB) radiograph was performed to determine the position of the ureteral stents after the operation. The distal coil of the stent was in the renal collecting system.

Three or six months after the end of the radiotherapy, the ureteroscope was placed into the ureter along with the ureteral orifice, and the ureteral lumen was checked. If there was no obvious stricture, we chose to remove ureteral stents. If the lumen of the ureter was obviously narrow, the ureteral stents were replaced.

#### Radiotherapy

Positron Emission Tomography—Computed Tomography (PET/CT) System (Discovery ST Elite‐Performance, GE Healthcare, USA) and the Elekta Synergy linear accelerator (Elekta, Crawley, UK) were selected in this study. Three-dimensional conformal radiotherapy (3D-CRT) with 6 MV X-ray photons was used for standard pelvic external beam radiation. The external dose was 45–50 Gy, and the daily divided dose was 1.8–2.0 Gy. The radiation therapy was performed once a day, 5 days a week.

The areas of pelvic external beam radiation included 3–4 cm or higher above the vaginal stump, parauterine tissues, internal iliac lymph nodes, external iliac lymph nodes, obturator lymph nodes, presacral lymph nodes, and other lymph node drainage areas. If the lymph node was metastasized, the radiation field was expanded accordingly, including the common iliac lymph nodes and para-aortic lymph nodes. If the lower third of the vagina was invaded, the radiation field was expanded to the bilateral inguinal lymph nodes.

### Follow-up

#### Diagnosis of ureter stricture

Patient follow-up was conducted through hospital visits or telephone calls starting from the day of radiation therapy ends. The first reassessment of urologic ultrasound was conducted within 3 months after radiotherapy, and the subsequent reexaminations were recommended every 6 months. The time of follow-up was at least 6 months. Considering the periodic replacement of the inside stent for stent group, ureteroscope with or without reaching the renal pelvis through ureteral or ureteral with or without stricture could be directly observed. There was not stent in vivo for the control group, ultrasonography could reveal ureter stricture and hydronephrosis due to its convenience, high accuracy, non-invasiveness and safety. Thus, ureteropyeloscopy was performed on the patients in stent group, while urologic ultrasound was conducted on the patients in control group. If hydroureteronephrosis was confirmed using urologic ultrasound, further ureteropyeloscopic examination was necessary.

In the stent group, the ureteroscope was checked 3 months after completing the radiation therapy. The diagnosis of ureteral stricture was confirmed if ureteroscope cannot reach the renal pelvis through ureteral, the lumen was narrowed, stents were replaced due to low back pain, fever after removing the stents, and/or hydronephrosis was detected by color doppler ultrasound. In the control group, the patients with hydronephrosis were diagnosed with ureteral strictures following urinary tract ultrasound and ureteropyeloscopic examinations. In parallel, the relapse must be confirmed by computed tomography (CT).

### Statistical analysis

All data were analyzed by SPSS software version 25.0, and were expressed as mean ± standard deviation ($\overline{X}$ ± S) or median (minimum, maximum). Meanwhile, the enumeration data were presented as a percentage or rate (n, %). T-test, rank-sum test or χ^2^ test was used to compare the differences between the two groups. Statistical significance level was set at *P* < 0.05.

## Results

### Comparison of the baseline data between the two groups

After screening through the inclusion and exclusion criteria, a total of 55 patients were eligible for this study. The stent group consisted of 26 patients (average age = 51.38 ± 8.75 years), while the control group consisted of 29 patients (average age = 51.69 ± 11.64 years). The number of patients with squamous-cell carcinoma (n = 23, 88.5%) was the highest in the stent group, followed by adenocarcinoma (n = 2, 7.7%) and adenosquamous carcinoma (n = 1, 3.8%). Meanwhile, squamous-cell carcinoma (n = 25, 86.2%) was the most common type of cervical cancer in the control group, followed by adenosquamous carcinoma (n = 3, 10.3%) and adenocarcinoma (n = 1, 3.4%). However, no significant differences in baseline data were observed between the the two groups (*P* > 0.05; Table 1).

**Table 1:** Comparison of the baseline data between the two groups ($\overline{\user2{X}}$ ± S)

Clinical features	Stents group	Control group	T/Z/χ.^2^	*P*
	(n = 26)	(n = 29)		
Age (years)	51.38 ± 8.75	51.69 ± 11.64	0.109	0.914
*Surgery type (n, %)*			2.988	0.224
Radical hysterectomy	0 (0.0%)	1 (3.4%)		
Radical hysterectomy + pelvic lymphadenectomy	4 (15.4%)	9 (31.0%)		
Radical hysterectomy + pelvic and para-aortic lymphadenectomy	22 (84.6%)	19 (65.5%)		
*FIGO clinical stage (n, %)*			0.906	0.636
Stage I	14 (53.8)	18 (62.1)		
Stage II	7 (26.9)	8 (27.6)		
Stage III	5 (19.2)	3 (10.3)		
*Pathological type (n, %)*			1.257	0.533
Carcinoma, squamous cell	23 (88.5)	25 (86.2)		
Adenocarcinoma	2 (7.7)	1 (3.4)		
Carcinoma, denosquamous	1 (3.8)	3 (10.3)		
Radiotherapy time (days)	38.50 ± 7.49	35.48 ± 5.08	1.765	0.083
Creatinine (µmol L^−1^)	62.18 ± 6.45	58.28 ± 7.88	1.995	0.051
Urea nitrogen (mmol L^−1^)	4.00 ± 1.13	4.10 ± 1.05	0.342	0.734
Glomerular filtration rate (ml min^−1^)	91.13 ± 13.70	99.05 ± 15.88	1.971	0.054
Urinalysis specific gravity	1.01 ± 0.01	1.01 ± 0.01	0.865	0.391

### Ureteral stricture

Three months after the end of radiotherapy, 12 patients in the stent group (46.2%) had ureteral stricture, of which 4 were unilateral and 8 were bilateral. There was a total of 20 units and the total incidence of ureteral strictures in stent group was 38.5%. Ten cases of ureteral stricture occurred in the control group, and the incidence of ureteral stricture was 34.5%. Of them, 5 patients were unilateral and the remaining patients were bilateral. There was a total of 15 units and the total incidence of ureteral strictures in control was 25.9%. However, there were no significant differences in the incidence rates of ureteral stricture and ureteral strictures between the two groups (*P* > 0.05; Table 2).

**Table 2:** Comparison of the occurrence of ureteral stricture between the two groups

Items	Stents group (n = 26)	Control group (n = 29)	χ^2^	*P*
*Stricture (n, %)*			0.778	0.378
Yes	12 (46.2)	10 (34.5)		
No	14 (53.8)	19 (65.5)		
*Stricture (units, %)*			2.006	0.157
Yes	20 (38.5)	15 (25.9)		
No	32 (61.5)	43 (74.1)		

Six months after the end of the radiotherapy, 13 patients in the stent group (50.0%) had ureteral stricture, of which 5 were unilateral and 8 were bilateral. There was a total of 21 units and the total incidence of ureteral strictures in stent group was 40.4%. Ten cases of ureteral stricture occurred in the control group, and the incidence of ureteral stricture was 34.5%. Of them, 5 patients were unilateral and the remaining patients were bilateral. There was a total of 15 units and the total incidence of ureteral strictures in control was 25.9%. However, there were no significant differences in the incidence rates of ureteral stricture and ureteral strictures between the two groups (*P* > 0.05).

### Complications related to stent placement

Complications related to indwelling ureteral stents in the stent group were urinary tract infections (n = 8, 30.8%), lumbar pain (n = 8, 30.8%), fever (n = 9, 34.7%), bladder irritation (n = 10, 38.5%) and hematuria (n = 5, 19.2%). There were no other complications such as stent displacement and stone formation. Complications related to indwelling ureteral stents in the control group were urinary tract infections (n = 1, 3.4%), lumbar pain (n = 3, 10.3%), fever (n = 4, 13.8%), bladder irritation (n = 4, 13.8%) and hematuria (n = 4, 13.8%). The urinary tract infections and bladder irritation between the two groups were statistically significant (*P* = 0.006 and *P* = 0.036); while the other complications were not statistically significant (*P* = 0.070, *P* = 0.092, and *P* = 0.586; Table 3).

**Table 3:** Comparison of the complications between the two groups

Items	Stents group (n = 26)	Control group (n = 29)	χ.^2^	*P*
*Fever (n, %)*			3.293	0.070
Yes	9 (34.6)	4 (13.8)		
No	17 (65.4)	25 (86.2)		
*Lumbar pain (n, %)*			3.574	0.092
Yes	8 (30.8)	3 (10.3)		
No	18 (69.2)	26 (89.7)		
*Urinary tract infections (n, %)*			7.477	0.006
Yes	8 (30.8)	1 (3.4)		
No	18 (69.2)	28 (96.6)		
*Bladder irritation (n, %)*			4.396	0.036
Yes	10 (38.5)	4 (13.8)		
No	16 (61.5)	25 (86.2)		
*Hematuria (n, %)*			0.296	0.586
Yes	5 (19.2)	4 (13.8)		
No	21 (80.2)	25 (86.2)		

### Stent removal and follow-up

The definitions of stent failure were failure to alleviate predominant symptoms or signs, irritative symptoms by ureteral stents, the need for additional therapies, or unexpected or early changing ureteral stents (normal stent placement was 3 months).

At 3-month follow-up, 16 patients in the stent group had successfully stent removal (2 patients had a stent replacement due to fever or hydronephrosis after stent removal). Ureteroscopic assessment showed that the ureteral lumen was significantly narrowed in 10 patients, and the stents were successfully replaced. A total of 12 patients failed to remove stents, and the success rate of stent removal was 53.8%. At 6-month follow-up, the 12 patients who were failed to remove stents still had ureteral narrowing, and the stents were continued to be replaced. In the 14 patients who were successfully removed stents, one patient had stent replacement due to ureteral stricture.

## Discussion

Among the most common cancers that affect women in the world, cervical cancer ranks fourth for both incidence and mortality [8]. According to the global cervical cancer report in 2018, there were 570,000 new cases and 311,000 deaths annually, which seriously threatened the health and quality of life of women around the world [9].

Surgery, radiotherapy and surgery combined with radiotherapy/ chemoradiotherapy are currently the three main treatment options for cervical cancer patients. However, surgery may damage the ureter and cause ureteral stricture. According to the previous reports, the incidence of ureteral injury caused by gynecological surgery and radical hysterectomy ranged from 0.083% to 0.40% and 0.72% to 1.4%, respectively [10–13]. High-risk factors for ureteral injury included pelvic anatomical abnormalities, fibrosis, pelvic surgery history, radiotherapy history, ureteral abnormalities, malignant tumors, etc. [14, 15]. Some physicians choose to insert ureteral stents before the operation [16, 17]. They believe that the preoperative stent is helpful for the intraoperative identification of the ureters and reducing the risk of ureteral injury during the operation. At the same time, if the ureter was injured, the ureteral stent could be used for early diagnosis, prognosis and treatment monitoring.

According to the National Comprehensive Cancer Network (NCCN) guidelines [1], postoperative radiotherapy is highly recommended for cervical cancer patients with pathological risk factors. Radiotherapy has a definite effect on cervical cancer, but the incidence of adverse reactions after this treatment is very high, including ureteral stricture and radiation cystitis [2–4]. Since the middle and lower part of the ureter is on the same plane as the pelvis, it is easily affected by radiotherapy. Therefore, ureteral stricture often occurs in the middle and lower part of the ureter after radiotherapy. Welk et al. [18] retrospectively analyzed a Canadian database of adult cervical cancer patients from 1994 to 2014, and found that the incidence of ureteral stricture after radiotherapy was 16%, which was higher than the surgery and radiation group (11%) and surgery alone group (5%). Elliott et al. [19] also reported that the incidence of ureteral stricture in cervical cancer patients treated with radiotherapy after radical resection was lower than that of patients treated with radiotherapy alone. The occurrence of ureteral stricture is associated with the total dose of radiotherapy, a single dose of radiotherapy, the length of radiotherapy, daily radiation dose, and tumor stage [20]. Gillette et al. [20] found that the incidence of ureteral strictures was significantly related to the amount of radiation exposure in animal experiments. As the radiation dose increases, the degree of ureteral damage becomes more severe. Van Kampen et al. [21] showed that the incidence of ureteral strictures after ureteral radiotherapy was positively correlated with the area receiving radiotherapy in dogs. After radiotherapy, the incidence of ureteral stricture in cervical cancer patients was proportional to the total amount and single dose of radiotherapy [2].

Lobo et al. [22], report incidences diagnosed at 3 years. Besides, these rates, increase significantly, specially, between the 3rd and the 5th year. It is common that radiotherapy-induces ureteral and urethral strictures are reported over the years. Li et al. [23] even categorize short-term strictures when they are diagnosed within the first 12 months after the last dose of radiotherapy and long-term after 12 months. Even the publication cited by the authors, by McIntyre et al. from 1995 [3] reports an increasing incidence of radiotherapy induced strictures within 5 to 20 years of follow-up [24, 25]. Early detection of radiogenic ureteric stenosis is not easy, as the mean latency time for manifestation of the condition is 16.8 years [25]. 3-month or even 6-month follow-up may bias our results, but considering relatively rare the volume of eligible patients, the difficulty of follow-up etc., we only chose the relatively short follow-up. We will continue follow-up these patients in the future.

At present, there is no consensus on the ideal treatment method for ureteral stricture in patients with malignant tumors after radiotherapy [26, 27]. In view of its poor prognosis, the median survival time of cancer patients with ureteral stricture is only 5 months. Among patients with Eastern Cooperative Oncology Group (ECOG) score ≥ 2 and/or 4 or more malignant tumor-related events, the 1-, 6- and 12-month survival rates are even more worse [28]. Ureteral stricture after radiotherapy is one of the challenging problems in urology [29]. Thus, it is of great importance to prevent ureteral stricture in cancer patients treated with radiotherapy.

In the course of radical surgery for cervical cancer, whether preoperative ureteral stent placement can prevent the occurrence of ureteral stricture is still very controversial. Hwang et al. [30] demonstrated that preloaded ureteral stents could identify and reduce ureteral damage in patients treated with laparoscopic radical hysterectomy. It is highly recommended that ureteral stents should be indwelled before surgery. Some scholars reported that the incidence of complications related to stent placement, such as ureteral perforation, was low after reserving ureteral stents or catheters, and they believed that this approach was safe and reliable [31]. However, there is still a lack of large studies to support the hypothesis that preoperative stent placement can prevent the risk of ureteral injuries. In a prospective trial conducted by Chou et al. [32], the patients who underwent grade 3 or 4 gynecological surgery were divided into reserved stent group and unreserved stent group. The results showed that the incidence rates of ureteral injury were 1.2% and 1.09% in the reserved and unreserved stent groups, respectively, with no statistically significant difference (*P* > 0.05). They clearly stated that stent placement could not reduce the incidence of ureteral injury in patients with gynecological cancer.

At the time of adjuvant radiotherapy after radical resection, whether preoperative ureteral stents can prevent the occurrence of ureteral stricture is also very controversial. Demanes et al. [33] studied 289 patients who were diagnosed as cervical cancer without ureteral stricture. There were 11 ureteral stricture cases in 255 patients without indwelled ureteral stents before brachytherapy, and no ureteral stricture case was found in 34 patients with indwelled ureteral stents. Thus, their findings indicate that the placement of ureteral stents can reduce the radiation dose received to the ureter and prevent stricture during radiotherapy. For pelvic external beam radiation, whether preoperative ureteral stents can reduce the occurrence of ureteral stricture has yet to be investigated. The results of this study showed that 12 (46.2%) and 10 (34.5%) cases of ureteral stricture occurred in the stent and control groups, respectively, at 3 months after radiotherapy. The difference was not statistically significant (*P* > 0.05), which indicated that preoperative ureteral stents could not reduce the occurrence of ureteral strictures after radiotherapy.

In addition to the medical burden, indwelling stents can also lead to unfavourable complications, such as stone formation, lumbar pain, urinary tract infections, stent displacement, stent stricture, stent blockage, fever and others, which severely reduce the quality of life of the patients. The rate of readmission due to complications was up to 19% [34–36]. Stent stone rate was only about 2% when the ureteral stent was used to manage benign ureteral stricture, while the rate ranged from 38% in malignant ureteral stricture [37, 38]. The majority of chronic indwelling polymer ureteral stents are routinely changed every 3 to 6 months. In the case of stent stone, the changing time may be shorter. Therefore, patients will be readmitted to the hospital due to those complications during follow-up.

Ureteral stent indwelled may be faced with stent failure. The definitions of stent failure were failure to alleviate predominant symptoms or signs, irritative symptoms by ureteral stents, or the need for additional therapies. It usually occurs within 6 ~ 12 months after the stent first placement. Frequent urination, urinary urgency, dysuria, and abdominal pain were the subjective evidence, which abdominal pain was the most frequent complaint. Maenwhile, the objective evidence included recurrent/persistent hydronephrosis, persistent rising serum creatinine, and stent stone formation etc.. The most common manifestation was elevated serum creatinine levels [39, 40].

Some scholars believed that unexpected or early changing ureteral stents (most polymer stent is 3–6 months, metallic stent is 1 year) was defined as stent failure [41].

During follow-up, we found that cervical cancer patients with retained ureteral stents had a difficulty in removing the stents. At 3-month follow-up, a total of 12 patients failed to remove stents, and the success rate of stent removal was only 53.8%, which was much lower than the previously reported values [29, 42]. The reason may be that this study was conducted on a retrospective basis. Our study possessed a highly selective for patients, that is, patients who cannot cooperation, or those with incomplete data or lost to follow-up were excluded. At 6-month follow-up, the 12 patients who were failed to remove stents had ureteral narrowing, and the stents were replaced. In the remaining 14 patients who had successful stent removal, only one patient underwent stent replacement due to ureteral stricture.

## Limitations of the study

Nevertheless, there were some limitations to the study. Firstly, the sample size of this study was relatively small. Therefore, expanding the sample size is needed in future studies. Secondly, the study was a single center retrospective analysis, which had a short-term diagnosis and follow-up. Cervical cancer patients treated with radiotherapy after radical resection might have a prolonged course of the disease, complicated conditions and unknown records. All of these can lead to human bias. Thirdly, only common ureteral stents were investigated in this study. Because common ureteral stents are easily squeezed, deformed and low compression resistance, there is a lack of comparison between common ureteral stents and other types of stents. Thus, a large-scale, multi-center, long-term prospective study is needed to confirm our data. Besides, a prospective comparison between different ureteral stents is required to be carried out.

## Conclusion

In summary, preoperative stent placement may not reduce the incidence of ureter stricture in cervical cancer patients treated with adjuvant radiotherapy after radical resection. The success rate of stent removal is low, the stent needs to be replaced regularly, and the complications related to indwelling ureteral stents may occur at any time. Thus, preoperative stent placement should be cautious for the clinical management of cervical cancer patients treated with postoperative radiotherapy.

## Data Availability

The datasets used and/or analysed during the current study are available from the corresponding author on reasonable request.
